# Characterization of a wheat stable QTL for spike length and its genetic effects on yield-related traits

**DOI:** 10.1186/s12870-024-04963-3

**Published:** 2024-04-17

**Authors:** Hongke Ding, Chenyang Wang, Yibiao Cai, Kai Yu, Haibo Zhao, Faxiang Wang, Xinyao Shi, Jiajia Cheng, Han Sun, Yongzhen Wu, Ran Qin, Cheng Liu, Chunhua Zhao, Xiaohui Sun, Fa Cui

**Affiliations:** 1 Key Laboratory of Molecular Module-Based Breeding of High Yield and Abiotic Resistant Plants in Universities of Shandong, College of Agriculture, Ludong University, Yantai, 264025 China; 2 Yantai Agricultural Technology Extension Center, Yantai, 264001 China; 3 Crop Research Institute, Shandong Academy of Agricultural Sciences, Jinan, 250100 China; 4 Yantai Academy of Agricultural Sciences, Yantai, Shandong 265500 China

**Keywords:** Wheat (*Triticum aestivum* L.), Spike length, QTL, Molecular markers, Genetic effect analysis, Breeding selection effect

## Abstract

Spike length (SL) is one of the most important agronomic traits affecting yield potential and stability in wheat. In this study, a major stable quantitative trait locus (QTL) for SL, i.e., *qSl-2B*, was detected in multiple environments in a recombinant inbred line (RIL) mapping population, KJ-RILs, derived from a cross between Kenong 9204 (KN9204) and Jing 411 (J411). The *qSl-2B* QTL was mapped to the 60.06–73.06 Mb region on chromosome 2B and could be identified in multiple mapping populations. An InDel molecular marker in the target region was developed based on a sequence analysis of the two parents. To further clarify the breeding use potential of *qSl-2B*, we analyzed its genetic effects and breeding selection effect using both the KJ-RIL population and a natural mapping population, which consisted of 316 breeding varieties/advanced lines. The results showed that the *qSl-2B* alleles from KN9204 showed inconsistent genetic effects on SL in the two mapping populations. Moreover, in the KJ-RILs population, the additive effects analysis of *qSl-2B* showed that additive effect was higher when both *qSl-2D* and *qSl-5A* harbor negative alleles under LN and HN. In China, a moderate selection utilization rate for *qSl-2B* was found in the Huanghuai winter wheat area and the selective utilization rate for *qSl-2B* continues to increase. The above findings provided a foundation for the genetic improvement of wheat SL in the future via molecular breeding strategies.

**Supplementary Information:**

The online version contains supplementary material available at 10.1186/s12870-024-04963-3.

## Key message

A major stable QTL for spike length was identified in wheat, and its genetic effect on yield related-traits as well as its potential use value in molecular breeding programs were characterized.

**Supplementary Information:**

The online version contains supplementary material available at 10.1186/s12870-024-04963-3.

## Introduction

Wheat (*Triticum aestivum* L.) is a major food crop that is grown across the globe and increasing its yielding potential is important if world hunger levels are to be alleviated [1, 2]. Continuous population growth and the reduction in global arable land mean that it is particularly important to cultivate new wheat varieties with high and stable yield potentials [3].

Spike length (SL) is an important component of spike morphology in wheat and has an important influence on yield [2, 4]. Previous reports have indicated that SL was usually closely correlated with spike compactness (SCN), thousand kernel weight (TKW), and grain yield per plant (GYPP) [5–9]. In addition, Jantasuriyarat et al. [10] reported that SL was positively correlated with spikelet number per spike (SNPS) and that an allele from W7984 could have simultaneous genetic effects on both SCN and SL. Moghaddam et al. [11] reported that SL was positively correlated with shoot biomass, straw biomass per plant, and grain yield. Donmez et al. [12] reported that SL was positively correlated with harvest index, aboveground biomass, and grain yield after evaluating grain yield related traits. Therefore, identifying and verifying the genetic loci that control SL is crucial to the genetic improvement of wheat yields [3, 13].

Advances in molecular biology and quantitative genetics have led to the mapping of genes and numerous quantitative trait loci (QTLs) controlling SL on almost all the 21 chromosomes in wheat [13–19]. For example, the *Q* gene located on chromosome 5A allows the grain to be free-threshed and is closely related to SL, plant height (PH), and SCN [20, 21]. In addition, Zhang et al. [22] reported that *TaAIRP2-1B* was a regulator of SL in wheat; Ji et al. [23] mapped *QSl.cib*-5A on chromosome 5A and reported that it explained 7.88–26.6% of the phenotypic variation; Gao et al. [24] mapped *QSL.caas-4AS* and *QSL.caas-4AL.1* on chromosome 4A and demonstrated that they individually explained 4.5–12.3% and 6.8–11.9% of the phenotypic variation, respectively; and Yu et al. [2] mapped 13 QTLs on chromosomes 1A, 1B, 3D, 4A, 5A, 5D, 6A, 7A, and 7B and showed that they individually explained 3.3–25% of the phenotypic variation. A QTL cluster associated with SL was also mapped onto chromosome 5A by Zhai et al. [25]. The abovementioned QTLs for SL have led to the breeding of wheat varieties with high and stable yields via molecular breeding technology. However, few studies have analyzed the genetic and breeding selection effects of the genes/QTLs related to SL.

Here, a major and stable quantitative trait locus (QTL) for SL, *qSl-2B*, was detected in multiple environments in a Kenong 9204 × Jing 411 derived recombinant inbred line (KJ-RIL) and was mapped to the 60.06–73.06 Mb region on chromosome 2B (based on the genome assembly for Kenong 9204, WheatOmics, http://202.194.139.32/). To further clarify its breeding potential, we analyzed the genetic and breeding selection effects of *qSl-2B* using the KJ-RIL population and a natural mapping population consisting of 316 breeding varieties/advanced lines. In addition, an InDel molecular marker, *2B-ID63*, that is closely linked to *qSl-2B* was developed and the results showed that it improved wheat development based on molecular marker-assisted selection. Our results provided a foundation for the genetic improvement of wheat SL-related traits in the future and provided more molecular markers for molecular breeding programs that aimed to improve the yield potential of wheat.

## Materials and methods

### Plant materials and field trials

A total of 188 recombinant inbred line (RIL) populations, referred to “KJ-RILs”, derived from a cross between Kenong 9204 (KN9204) and Jing 411 (J411), were used in this study. Among them, KJ129 was not used in this study because a large number of the SNP markers were not present in the genotype. KN9204, J411, and the KJ-RILs were provided by the Center for Agricultural Resources Research, Institute of Genetics and Developmental Biology, Chinese Academy of Sciences. The parents and the KJ-RIL populations were grown in ten different environments for phenotype evaluation. The low nitrogen (LN) environments were located in the following places: 2011–2012 in Shijiazhuang (E1-LN), 2012–2013 in Shijiazhuang (E3-LN), 2012–2013 in Beijing (E5-LN), 2012–2013 in Xinxiang (E7-LN), and 2013–2014 in Shijiazhuang (E9-LN). The high nitrogen (HN) environments were located in the following places: 2011–2012 in Shijiazhuang (E2-HN), 2012–2013 in Shijiazhuang (E4-HN), 2012–2013 in Beijing (E6-HN), 2012–2013 in Xinxiang (E8-HN), and 2013–2014 in Shijiazhuang (E10-HN). Detailed information regarding soil nitrogen content, experimental design, etc., can be found in our previous reports [4, 26–30]. The natural mapping population consisted of 316 breeding varieties/advanced lines and was planted in four environments for phenotype evaluation: 2020–2021 in Shijiazhuang, 2020–2021 in Weifang, 2020–2021 in Pulagu, and 2020–2021 in Yantai, China. A randomized block design with two replicates was used in each of the four environments. A three-row plot with rows that were 1.5 m long and 0.25 m apart was used, and 30 seeds were planted in each row. All experimental fields were routinely managed according to local standards.

### Data collation and analysis

Based on the methods described in Cui et al. [16, 31], nine traits, SL, PH, grain length (GL), GYPP, spike number per plant (SNPP), TKW, SCN, kernel number per spike (KNPS), and SNPS, were measured in the natural mapping population and the KJ-RILs. QGAStation2.0 [32] was used to analyze the best linear unbiased estimators (BLUEs) for SL under LN (E1, E3, E5, E7, and E9) and HN (E2, E4, E6, E8, and E10) conditions and two datasets, BLUE-LN and BLUE-HN, were obtained. Both datasets were used in the QTL mapping analysis. Similarly, QGAStation 2.0 was used to analyze the BLUEs for yield-related traits in the natural mapping population grown in the four environments. A broad-sense heritability (*H*^2^) analysis was also performed using QGAStation 2.0. A one-way ANOVA and a phenotypic correlation analysis of SL with other traits were also performed using SPSS17.0 (https://spss-64bits.en.softonic.com/). The KJ-RIL and the natural mapping populations were used to perform an association analysis of the genotype and phenotypic data to estimate the genetic effect of *qSl-2B* on yield-related traits. FineBI 5.0 (https://www.finebi.com/product/fbi5) was used to analyze the selection effect of *qSl-2B* on the breeding process across the different regions and years.

### Genotype acquisition and QTL detection

The DNA from the 187 KJ-RILs and the 316 accessions in the natural mapping population was extracted using the CTAB method according to Stacey et al. [33] with minor modification. The genotypes of the KJ-RIL and the natural mapping populations were screened using the 660 K and 55 K SNP arrays, respectively. The physical position of each SNP in the KN9204 genome assembly (unpublished data) was obtained by a local BLAST analysis (ftp://ftp.ncbi.nlm.nih.gov/blast/execuTABLEs/release/). A high density linkage map containing 119566 loci has been previously released by Cui et al. [34] and was based on a linkage analysis of the KJ-RIL population. In the present study, the physical positions rather than the genetic positions of the markers were used in the QTL mapping analysis.

Quantitative trait loci detection was performed using the IciMapping V4.2 BIP module format (http://www.isbreeding.net) with a logarithm of odds (LOD) threshold based on 1,000 permutations, 1.0 Mb as the step size, a stepwise regression of *P* = 0.001, and an *I*-error of 0.05.

### Development of the InDel molecular markers

Based on a 10×genome resequencing of KN9204 and J411, a 51 bp insertion and deletion (InDel) polymorphic DNA sequence was identified at KN2B: 63085329 of the KN9204 genome. This was then used for polymorphic molecular marker development. PrimerServer in WheatOmics1.0 (http://202.194.139.32/) was used to design the polymorphic InDel PCR primers and the primer sequences are listed in Table S1. The amplification of InDel molecular markers in the KJ-RIL population and the linkage analysis indicated that this marker could be used as a diagnostic marker to identify *qSl-2B* in molecular breeding programs.

## Results

### Phenotypic analysis of spike length in the KJ-RILs

The phenotypic analysis of SL in the KJ-RIL mapping populations in the different environments are shown in Table 1. The SL mean in the KJ-RILs ranged from 6.69 cm to 9.55 cm and the absolute values of kurtosis and skewness were both less than 1, indicating that the phenotypic data for SL in the 187 KJ-RILs were approximately normally distributed. The phenotypic variability and the broad-sense heritability of SL in the KJ-RILs across the 10 environments were 8.17–10.22% and 0.52–0.92, respectively. Therefore, genetic factors were the main cause of phenotypic variation and SL was a typical quantitative trait that can be used in a QTL mapping analysis.

**Table 1:** Phenotypic analysis of SL in the 187 KJ-RILs in 10 environments

Environment	Parents		KJ-RIL							
	KN9204	J411	Min	Max	Mean	SD	CV (%)	skewness	kurtosis	H^2^
E1-LN	6.64	7.36	5.00	8.36	6.69	0.67	10.01	0.24	-0.22	0.57
E2-HN	6.60	6.95	4.70	8.90	6.81	0.70	10.22	0	0.15	0.52
E3-LN	8.72	8.89	6.86	11.07	8.74	0.76	8.70	0.10	0.37	0.81
E4-HN	9.58	9.82	7.49	12.57	9.55	0.78	8.17	0.13	0.79	0.80
E5-LN	7.71	8.32	5.87	10.45	8.12	0.74	9.11	0.13	0.40	0.85
E6-HN	8.26	8.94	6.03	10.58	8.64	0.81	9.36	0.10	0.15	0.92
E7-LN	8.07	8.25	6.57	10.80	8.14	0.76	9.38	0.35	0.64	0.65
E8-HN	8.15	8.67	6.20	11.19	8.13	0.78	9.60	0.43	0.73	0.69
E9-LN	7.15	7.91	5.40	10.30	7.47	0.74	9.91	0.33	0.81	0.78
E10-HN	7.69	7.97	5.80	10.10	7.76	0.73	9.41	0.33	0.46	0.78

SD: standard deviation. CV: coefficient of variation. *H*^2^: broad-sense heritability

The correlation analyses between SL and other yield-related traits under LN and HN conditions are shown in Table 2. The results showed that SL was significantly positively correlated with KNPS, SNPS, PH, and GL under both LN and HN conditions, but significantly negatively correlated with SCN. There was no significant correlation between SL and GYPP, SNPP, or TKW.

**Table 2:** Correlation analysis of SL and yield-related traits for the 187 KJ-RILs under LN and HN conditions

	Condition	KNPS	SNPS	PH	SCN	GL	GYPP	SNPP	TKW
SL	LN	0.26**	0.35**	0.38**	-0.55**	0.22**	0.07	-0.05	-0.04
	HN	0.24**	0.32**	0.39**	-0.58**	0.15*	0.12	0	-0.02

SL: spike length, KNPS: kernel number per spike, SNPS: spikelet number per spike, PH: plant height, SCN: spike compactness, GL: grain length, GYPP: grain yield per plant, SNPP: spike number per plant, TKW: thousand kernel weight

*, Significant difference at *P* < 0.05; **, highly significant difference at *P* < 0.01

### QTL detection of qSl-2B

**Fig. 1:**
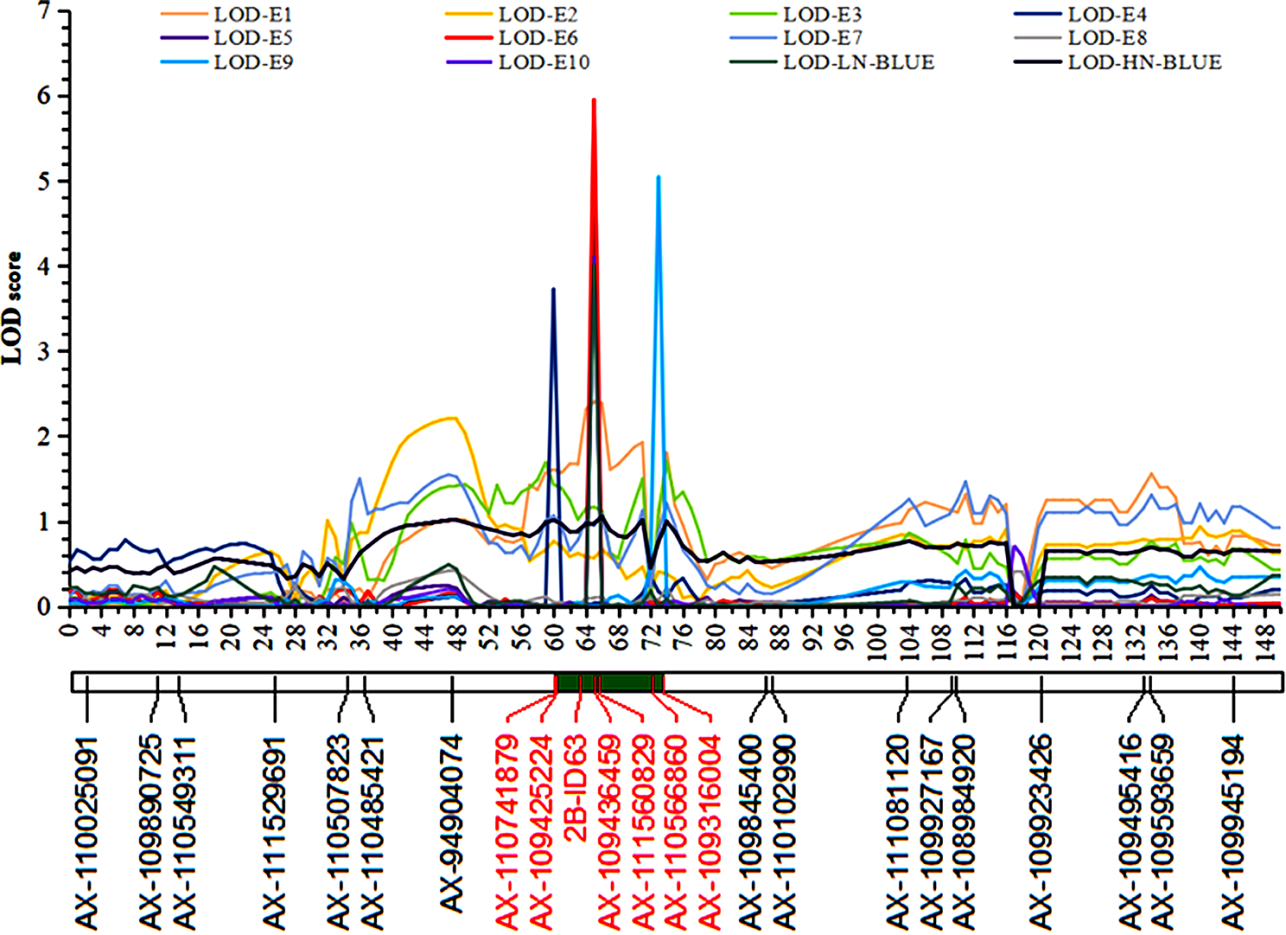
LOD value distributions for *qSl-2B* in the 10 environmental datasets and the 2 BLUE datasets. The abscissa shows the physical location of the SNP marker used for QTL positioning analysis in the reference genome of KN9204 and the ordinate is the LOD value corresponding to the different environments. The green segment is the estimated confidence region for *qSl-2B* and the markers in red were used in the subsequent genetic effect analysis

The QTL analysis showed that *qSl-2B* was consistently detected in the E4, E5, E6, E9, E10 as well as in the LN-BLUE and HN-BLUE datasets (Table 3; Fig. 1). It also explained 0.50–7.18% of the SL phenotypic variation in the KJ-RILs, with LOD values of 3.72–6.45. The favored alleles from KN9204 that augmenting SL, with the additive effect value ranging from 0.12 to 0.21. The physical information showed that *qSl-2B* was mapped within approximately 13 Mb at KN2B:60.06–73.06 Mb. Significantly, in five of the seven datasets shown in Table 3, *qSl-2B* was mapped to within 0.48 Mb (64.93–65.41 Mb) and was between the *AX-109436459* and *AX-111560829* SNPs. Furthermore, *qSl-2B* could be detected in three datasets representing the HN condition in Table 3 and the three datasets representing the LN condition, indicating that *qSl-2B* did not respond to nitrogen stress.

**Table 3:** The putative *qSl-2B* QTL was detected in the KJ-RILs across multiple environments by IciMapping v4.2

Environments	Position (Mb)^a^	Left Marker	Right Marker	LOD^b^	PVE (%)^c^	Add^d^
E4-HN	60.06	*AX-110741879*	*AX-109425224*	3.72	4.09	0.18
E5-LN	65.06	*AX-109436459*	*AX-111560829*	4.58	5.16	0.17
E6-HN	65.06	*AX-109436459*	*AX-111560829*	5.95	5.76	0.20
E9-LN	73.06	*AX-110566860*	*AX-109316004*	5.04	7.18	0.21
E10-HN	65.06	*AX-109436459*	*AX-111560829*	4.11	3.92	0.16
BLUE-LN	65.06	*AX-109436459*	*AX-111560829*	4.00	0.50	0.12
BLUE-HN	65.06	*AX-109436459*	*AX-111560829*	6.45	6.12	0.17

^a^The physical position of the LOD peak on KN9204 genome

^b^Logarithm of odds score

^c^Rates of the phenotypic variances explained by the QTL

^d^Additive efect of SL

### InDel molecular markers

**Fig. 2:**
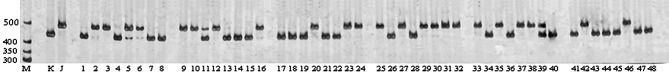
Electrophoresis results for the PCR products amplified by *2B-ID63* in the 187 KJ-RILs. M: DNA marker. K: KN9204. J: J411. Numbers 1–48 are the amplified products in the KJ-RILs

Based on the genome DNA sequence information for KN9204 and J411, a total of 308 InDels (larger than 5 bp) and 357 SNPs were identified within 5.46 Mb (59.95–65.41 Mb) on chromosome 2B. A close linkage PCR-based InDel molecular marker, *2B-ID63* (63,085,329), for *qSl-2B* was developed. It could clearly and stably amplify the 421 bp and 472 bp target fragments from KN9204 and J411, respectively, and these PCR product sizes were consistent with the DNA re-sequencing results for the two parental lines (Fig. 2). Therefore, *2B-ID63* could be used to further analyze the genetic effects of the spike length and yield traits for wheat and provide molecular markers for the genetic improvement of yield potential.

### Genetic effect of qSl-2B on yield-related traits in the KJ-RIL mapping population

**Fig. 3:**
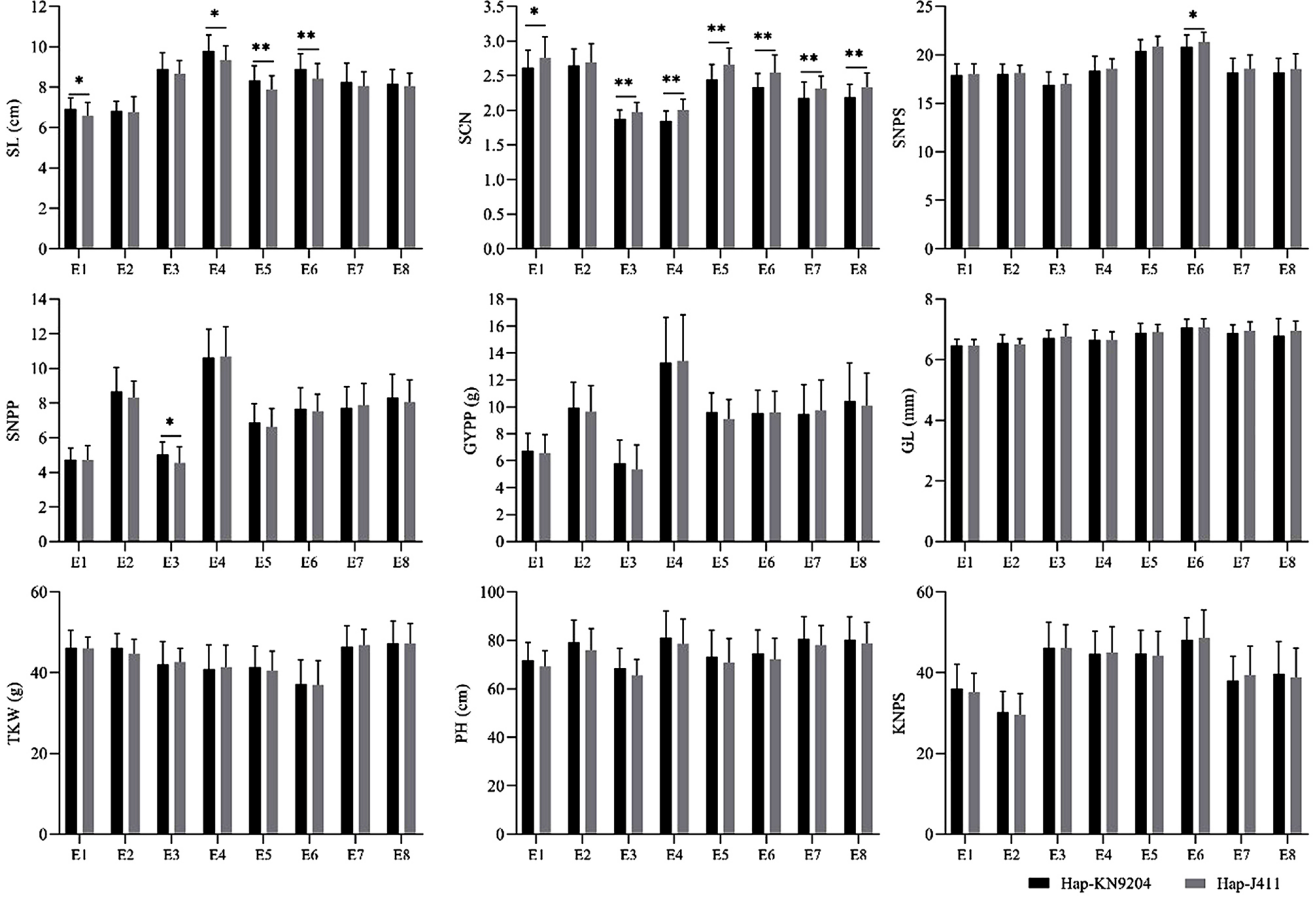
Genetic effects of *qSl-2B* on yield related traits in 8 of the 10 environments based on the KJ-RILs. SL: spike length, KNPS: kernel number per spike, SNPS: spikelet number per spike, PH: plant height, SCN: spike compactness, GL: grain length, GYPP: grain yield per plant, SNPP: spike number per plant, and TKW: thousand kernel weight. *, Significant difference at *P* < 0.05; **, highly significant difference at *P* < 0.01

The LOD peak position showed that *AX-110741879* (KN2B: 59.95 Mb), *AX-109425224* (KN2B: 60.19 Mb), *AX-109436459* (KN2B: 64.93 Mb), *AX-111560829* (KN2B: 65.41 Mb), *AX-110566860* (KN2B: 72.03 Mb), *AX-109316004* (KN2B: 73.42 Mb) and *2B-ID63* (KN2B: 63.09 Mb) was the close linkage markers for *qSl-2B*. Therefore, these markers were used to classify the 187 KJ-RILs into two groups. Since partial data for yield-related traits were missing in the E9 and E10 environments, the phenotypic data from the first 8 environments combined with genotypes were used to analyze for the genetic effect of *qSl-2B* on yield-related traits. The genotype identical to KN9204 was defined as *Hap-KN9204* and the genotype identical to J411 was defined as *Hap-J411*. The results showed that the excellent alleles from KN9204 could significantly increase SL in four of the eight environment datasets (Fig. 3), with an average increase of 5.31%, but could only significantly increase SNPP in one environment, with an average increase rate of 10.25%. However, it significantly decreased SCN in seven of the eight environments with an average decrease of 7.19%, significantly decreased SNPS in one of the eight environments with an average decrease rate of 2.59%. The *Hap-KN9204* allele had no significant effect on either GYPP, GL, TKW, PH or KNPS.

### Pyramiding effect analysis of qSl-2B under different genetic backgrounds

**Fig. 4:**
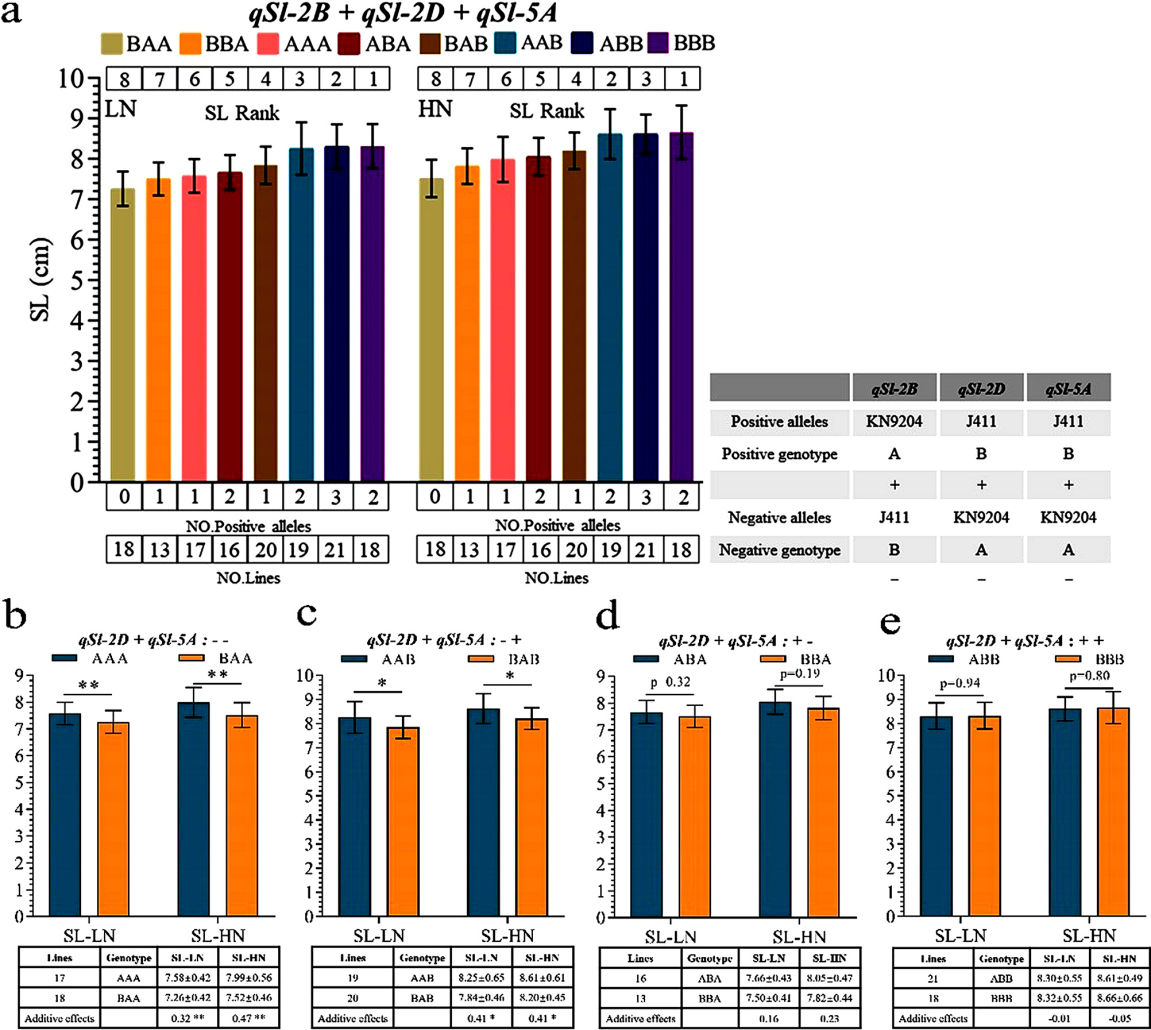
Pyramiding effect analysis of *qSl-2B* in different genetic backgrounds under LN and HN environments. (**a**) In the figure on the left, the Arabic numerals above each bar indicate the serial ID of the lines ranked according to SL value, from high to low SL. The Arabic numerals below each bar indicate number of positive alleles that increase SL. Arabic numerals at the bottom of each bar chart indicate the number of lines in each group. The figure on the right shows the origins of the positive and negative alleles for the three QTLs and the meaning of the code symbols. (**b**-**e**) The additive effects of *qSl-2B* in different genetic backgrounds under both HN and LN conditions. And the table at the bottom of in each bar chart shows the number of lines and the average SL (± SD) for each group. *, Significant difference at *P* < 0.05; **, highly significant difference at *P* < 0.01

In addition to *qSl-2B*, we also identified two stable additive QTLs for SL in the KJ population, namely *qSl-2D* and *qSl-5A* [30]. The positive alleles of the increased SL of *qSl-2D* and *qSl-5A* were from J411. The pyramiding effect analysis for these two QTLs and *qSl-2B* was performed. Based on the genotype of tightly linked markers of these three QTLs, the KJ-RILs mapping population was divided into 8 groups. The results showed that as the number of positive alleles increases under LN and HN, the length of the SL also tends to be increase longer, which confirms the significant additive effect of all three QTLs (Fig. 4a). Compared with the phenotype of BAA, the pyramiding of the two favorable QTL alleles (BBB) increased SL by 14.54% and 15.18% under LN and HN, respectively.

In order to further illustrate additive effect of *qSl-2B*, we divided the KJ-RILs population into four groups based on genotype of tightly linked markers of *qSl-2B*, *qSl-2D* and *qSl-5A*. The results showed that the additive effect of *qSl-2B* ranged from 0.47 to -0.05 (Fig. 4b-e). Of these, the additive effect of *qSl-2B* for SL was significant in the backgrounds of AA (− −) and AB (− +) (Fig. 4b-c). The additive effect was higher when both *qSl-2D* and *qSl-5A* harbor negative alleles under LN and HN. In the two backgrounds of BA (+ −) and BB (+ +), *qSl-2B* had no significant effect on SL (Fig. 4d-e). When both *qSl-2D* and *qSl-5A* harbor positive alleles, the additive effect was negative. These results suggest that the expression of *qSl-2B* might be repressed in these backgrounds to some extent.

### Genetic and breeding selection effects of qSl-2B based on the natural mapping population

Three SNP molecular markers, *i.e., AX-110426649* (TT/CC, KN2B: 60.21 Mb), *AX-110383086* (TT/CC, KN2B: 65.64 Mb) and *AX-108838208* (TT/CC, KN2B: 71.04 Mb), which were adjacent to the LOD peak position for *qSl-2B*, were used to genotype the 316 accessions in the natural mapping population (Table S2). The haplotype of *Hap-CC-CC-TT*, which was identical to KN9204, was defined as the excellent haplotype, *Hap-TT-TT-CC*, which was identical to J411, was defined as a non-excellent haplotype, and *Hap-CC-TT-TT*, *Hap-CC-TT-CC*, *Hap-TT-CC-TT*, *Hap-TT-CC-CC*, *Hap-CC-CC-CC*, and *Hap-TT-TT-TT* were defined as the recombinants. Heterozygous type is a line with a heterozygous genotype at any one to three of the three loci. Among the 316 accessions, the recombinants made-up the largest proportion, accounting for 34.81% of the total samples; the excellent haplotype and non-excellent haplotype accounting for 22.78% and 26.27%, respectively; and heterozygous type and missing data accounted for 5.70% and 10.44%, respectively (Table 4). These results indicated that *qSl-2B* was not fully utilized in traditional wheat breeding programs.

**Table 4:** Haplotype analysis of *qSl-2B* based on 316 accessions in the natural mapping population

Genotype	Quality	Proportion (%)
Excellent haplotype	72	22.78
Non-excellent haplotype	83	26.27
Recombinants	110	34.81
Heterozygous type	18	5.70
Missing data	33	10.44

**Fig. 5:**
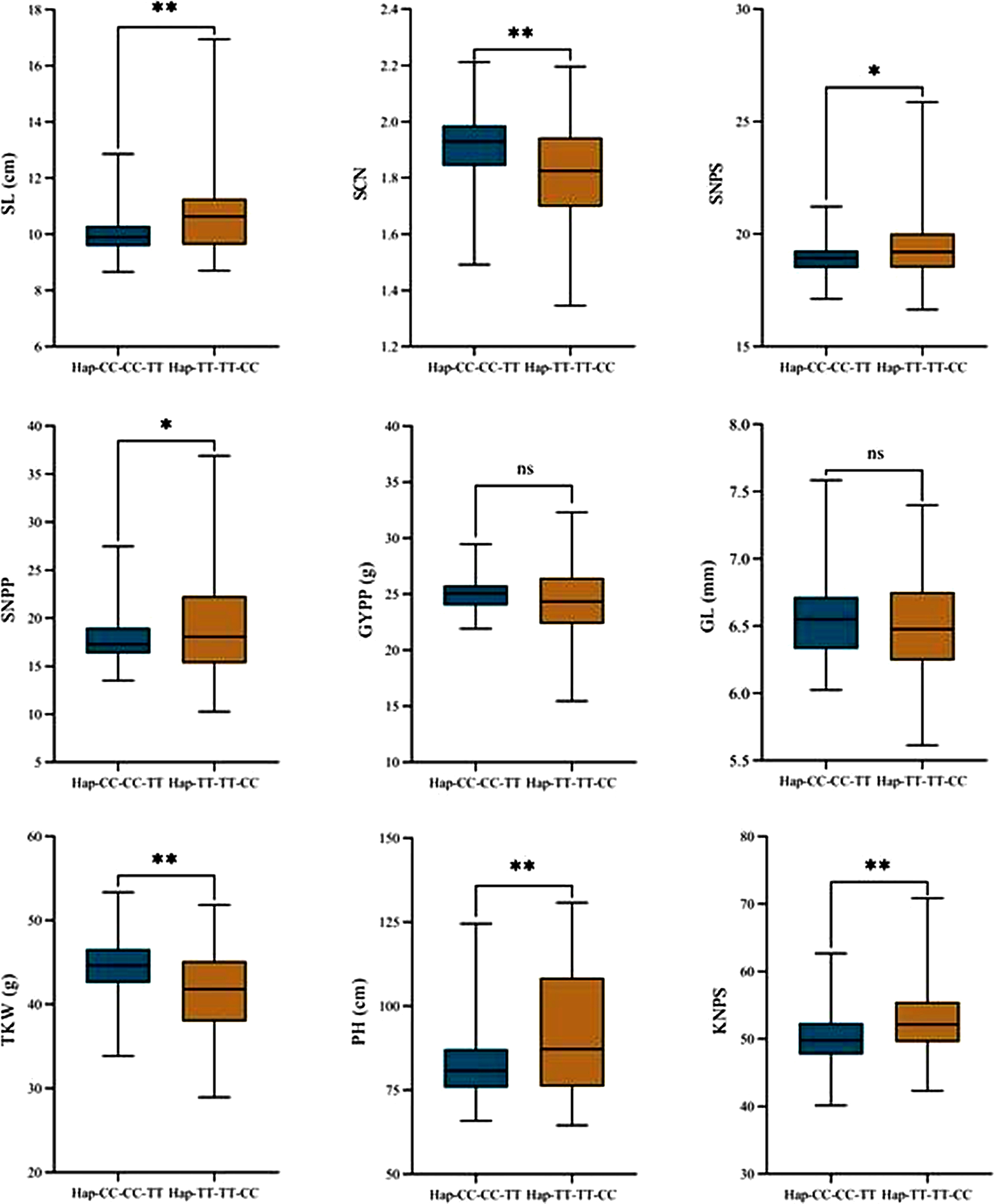
Genetic effects of *qSl-2B* on yield-related traits based on the 316 accessions in the natural mapping population. SL: spike length, KNPS: kernel number per spike, SNPS: spikelet number per spike, PH: plant height, SCN: spike compactness, GL: grain length, GYPP: grain yield per plant, SNPP: spike number per plant, TKW: thousand kernel weight. *, Significant difference at *P* < 0.05; **, highly significant difference at *P* < 0.01; ns: no significant difference

The genetic effects of *qSl-2B* on yield related traits were analyzed using the 316 accessions in the natural mapping population. The results showed that the excellent haplotype of *Hap-CC-CC-TT* could significantly increase TKW and SCN with average increase rates of 7.71% and 4.78%, respectively (Fig. 5). However, the *Hap-CC-CC-TT* haplotype significantly decreased SL, SNPS, SNPP, PH, and KNPS, with average decreases of 8.26%, 2.33%, 7.55%, 10.40%, and 5.40%%, respectively. However, its effects on GYPP and GL were not significant. Some of the results were inconsistent with those for the KJ-RIL population and may have been influenced by genetic background and environmental factors.

**Fig. 6:**
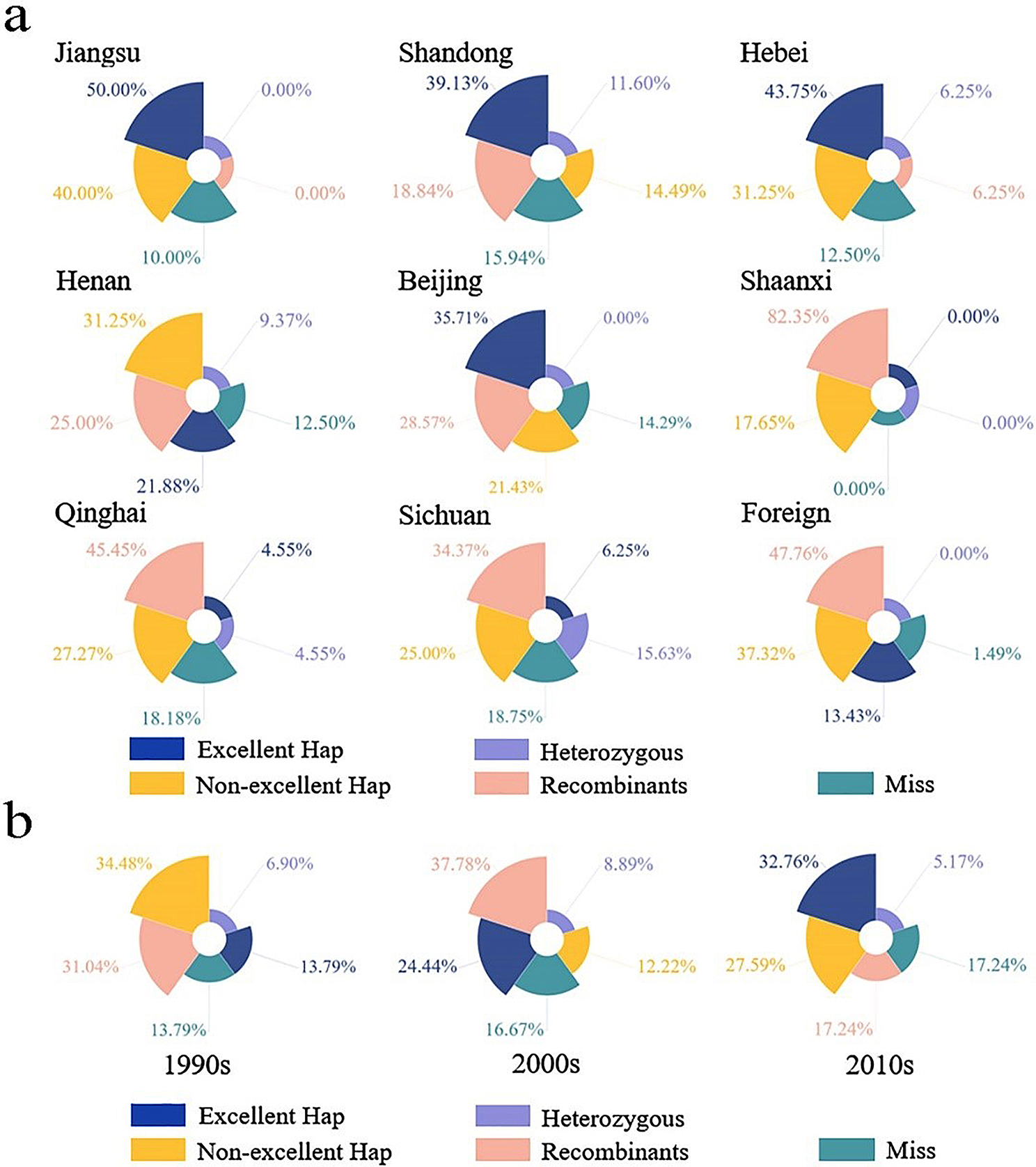
Analysis of the selection effect for *qSl-2B* haplotypes from the spatial (**a**) and time span (**b**) perspectives in breeding programs based on variety/advanced lines. Excellent Hap: Hap-CC-CC-TT; non-excellent Hap: Hap-TT-TT-CC; recombinants: Hap-CC-TT-TT, Hap-CC-TT-CC, Hap-TT-CC-TT, Hap-TT-CC-CC, Hap-CC-CC-CC, and Hap-TT-TT-TT; Heterozygous type is a line with a heterozygous genotype at any one to three of the three loci. Miss is a line where the haplotype data was missing. The corresponding radians of each haplotype in the chart were generated automatically by the software and have no practical significance

The breeding selection effects of *qSl-2B* at the spatio-temporal level were also analyzed based on geographical distribution using the natural mapping population (Fig. 6a). After discarding accessions with no detailed information, the 279 selected breeding varieties/advanced lines were divided into nine subgroups, which were those from Beijing, Hebei Province, Henan Province, Qinghai Province, Shandong Province, Shaanxi Province, Sichuan Province, Jiangsu Province, and countries/areas outside China. The results showed that the number of varieties with *Hap-CC-CC-TT* was highest in Jiangsu Province and Hebei Province, accounting for 50% and 43.75% of the total samples, respectively. In Shandong Province, Beijing and Henan Province, approximately 39.13%, 35.71% and 21.88% of the total samples, respectively, contained the excellent haplotype, but its utilization rates in Sichuan Province, Qinghai Province, and Shaanxi Province were lower, being 6.25%, 4.55%, and 0%, respectively. However, the utilization rates of the excellent haplotype were relatively low at 13.43%, but the non-excellent haplotype (*Hap-TT-TT-CC*) rate was 37.31% in the countries/areas that were outside China. The above results indicated that the utilization of *qSl-2B* considerably differed among regions/areas.

In terms of time span, the selection effect of *qSl-2B* was analyzed using 177 time-traceable varieties in the natural mapping population (Fig. 6b). In the 1990s, the non-excellent haplotype of *Hap-TT-TT-CC* was dominant and was present in up to 34.48% of the total samples, while the excellent haplotype of *Hap-CC-CC-TT* only accounted for 13.79% of the total samples. In the 2000s, the excellent haplotype proportion was 24.44%, while that of the non-excellent haplotype was 12.22% and those of the recombinants and the heterozygous type were 37.78% and 8.89%, respectively. In the 2010s, the excellent haplotype proportion was 32.76%, while that of the non-excellent haplotype was 27.59% and those of the recombinants and heterozygous type were 17.24% and 5.17%, respectively. These results indicated that wheat breeders have paid increasing attention to *qSl-2B* and applied its positive alleles to wheat breeding programs since the 1990s.

## Discussion

### Comparison of qSl-2B with that in previous studies

Spike length is a trait controlled by multiple genes and has important effects on wheat yield [35, 36]. Therefore, the identification and characterization of the QTL for SL is important when attempting to genetically improve yield potential based on a molecular breeding strategy. In this study, the SL of the KJ-RILs was evaluated in multiple environments under both LN and HN conditions. Among the five planting locations, except for the E7 and E8 in Xinxiang, the SL of the KJ-RILs was higher under HN than that under LN conditions. These findings indicated that SL was negatively affected by low nitrogen stress (Table 1). This result is consistent with previous reports, which suggested that nitrogen is an important nutrient for crop growth and development, including wheat [37–39].

Based on the genotypic data for the KJ-RILs, *qSl-2B* was mapped to the 60.06–73.06 Mb interval on chromosome 2B (based on the KN9204 genome assembly), and it was mapped to the KN2B: 64.93–65.41 Mb region in five of the 10 datasets, corresponding to the CS2B: 55.52–55.99 Mb section of the Chinese spring reference genome. To date, numerous QTLs for SL have been documented (http://wheatqtldb.net/yield_new.php) (Table S3) [9, 40–51]. Of these, Arif et al. [51] identified six QTLs controlling SL at 30.5 Mb, 144.65 Mb, 248.15 Mb, 546.61 Mb, 772.79 Mb, and 775.17 Mb on chromosome 2B, respectively. Under different soil moisture conditions, *QSl.cob-2B.1* was stably detected in three of the four environments in a DH mapping population by El-Feki et al. [48]. This QTL, which was localized within the CS2B: 47.53–88.83 Mb section, coincided with the physical position of *qSl-2B* in this study. Based on a genome-wide association analysis using a wheat 90 K iSelect SNP genotyping assay, Sun et al. [47] identified 129 significant SNPs that were associated with SL, among which *BS00022060_51* was located at CS2B: 52.7 Mb and therefore overlapped with *qSl-2B*. They also reported that it accounted for 4.4–21.0% of the phenotypic variation in this natural mapping population. The above results implied that *qSl-2B* could be identified in multiple mapping populations, which suggested that it may have important breeding applications.

**Fig. 7:**
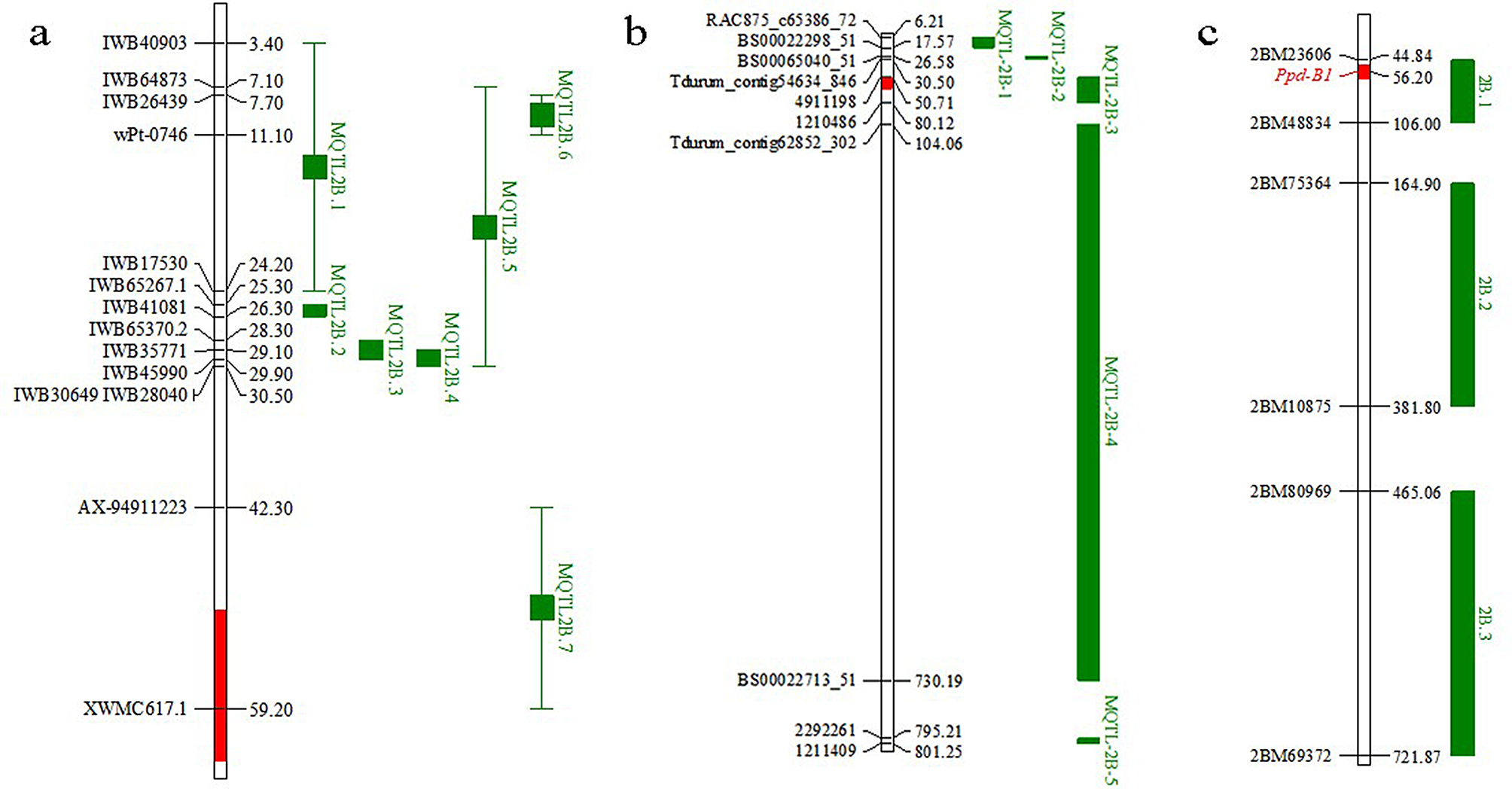
Meta-QTL and QTL clusters for spike-related traits on chromosome 2B reported in recent years. The figures a and b represent MQTL reported by Saini et al. [52] and Yang et al. [53], respectively. Figure c represents the QTL clusters reported by Zhang et al. [54]. The red segment on the three chromosomes is *qSl-2B* in the figure

According to meta-QTL analyses undertaken by recent studies [52–54], many other yield-related QTLs also coincide with the physical position of *qSl-2B* (Tables S3, S4, S5 and Fig. 7). A total of seven MQTLs on chromosome 2B were reported by Saini et al. [52] (Fig. 7a); of which *MQTL2B.7* was located at CS2B: 42.28–59.18 Mb and harbored QTLs for TKW, SL, PH, GYPP and other yield-related traits. *MQTL-2B-3* harbored QTL clusters for TKW, tiller number, KNPS, GYPP, and other yield-related traits that covered the CS2B: 50.71–80.12 Mb genome region (Fig. 7b) [53]. Zhang et al. [54] reported a QTL cluster called *2B.1* at CS2B: 44.8–106.00 Mb, which was associated with spikelet, GYPP, grain weight per tiller (GW), KNPS, and biomass (Fig. 7c). In addition, Zhang et al. [54] also found that the *Ppd-B1* gene (gene bank number DQ885765) is nearby this QTL cluster (2B.1) at 56.2 Mb, in which the copy number variation affects yield-related traits such as GYPP, TKW, KNPS, biomass, spikelet, PH, and keycard. The above results indicated that gene clusters or genes with pleiotropic effects on yield-related traits might existed near *qSl-2B* and that more attention should be paid to the key chromosomal segments in molecular breeding programs designed to improve yield potential.

### Genetic effect analysis of qSl-2B effects on yield related traits

Spike length is an important yield-related trait and is closely related to TKW, KNPS, GYPP, etc. Qu et al. [55] reported that SL was significantly positively correlated with TKW; Liu et al. [56] found that there were significant positive correlations among SL, SNPS, and KNPS; Guo et al. [57] reported that SL was significantly positively correlated with sterile spikelet number (SSN) and KNPS; and Li et al. [58] showed that SL and PH were significantly positively correlated with each other. In this study, SL was significantly positively correlated with SNPS, KNPS, GL, and PH and significantly negatively correlated with SCN (Table 2). These results were generally consistent with previous studies.

We also clarified the genetic effects of *qSl-2B* on other yield traits using the KJ-RIL and the natural mapping populations. In the natural mapping population, the genetic effect of *Hap-KN9204* on SL was opposite to that of the KJ-RILs with alleles from KN9204 increasing SL in the KJ-RILs, but decreasing SL in the natural mapping population (Figs. 3 and 5). Besides, *Hap-KN9204* had a positive effect on SNPP and negative effect on SCN, which is also completely reversed in natural mapping populations. The large confidence interval for the primary QTL mapping results and the high recombination frequency in the target region might account for this inconsistency. Fine mapping and map-based cloning of *qSl-2B* would verify this suggestion. In addition, QTL mapping based on a bi-parental mapping population could only identify the alleles that produce improvements compared to the two given parental lines, whereas in the natural mapping population, all or at least most of the possible allelic variation due to the target gene could be identified, which meant that the best alleles for a given gene could be identified. This might also account for the different results for the genetic effect of *qSl-2B* in the two different mapping populations. Future haplotype analyses of the target gene could be used to confirm this suggestion. In addition, *Hap-KN9204* had positive effect on TKW, and negative effect on PH and KNPS only in natural mapping populations. However, *Hap-KN9204* had a negative effect on SNPS in both the KJ-RIL and the natural mapping populations. The reason for this result may be that gene clusters and a unique gene underlying *qSl-2B* could exist in the target region.

### The qSl-2B selection utilization rate and future application potential

Traditional breeding technologies are mainly based on empirical selection for a specific phenotype, which is a slow process and the selection efficiency is generally low. Therefore, traditional breeding technologies cannot meet the demands associated with increasing wheat consumption through the genetic improvement of wheat yield potential. The 5G breeding approach will bring much-needed disruptive changes to crop improvement [59]. The key genes and close linkage molecular markers are very important when using the 5G breeding approach. To evaluate the potential application value of *qSl-2B* in future molecular breeding programs, we analyzed the selective utilization rate for *qSl-2B* in different regions and over different time periods. The results showed that the excellent *Hap-CC-CC-TT* haplotype from KN9204 was selected for use in some regions, but there were large differences among regions. In the regions outside China, the selective utilization rate for *Hap-CC-CC-TT* was low, indicating that breeders in countries outside China should pay more attention to this QTL. In China, *Hap-CC-CC-TT* selection was higher in Jiangsu, Shandong, and Hebei provinces, and Beijing particularly in the Huanghuai winter wheat area; but was lower in Henan Province, Beijing. Shanxi, Qinghai, and Sichuan. Different regions have different climates, soils, and other growth conditions and the focus associated with breeding for the genetic improvement of yield potential may vary. These variations might account for the large differences in the *qSl-2B* selection and utilization rates.

The selection and utilization of the excellent *Hap-CC-CC-TT* haplotype increased over time. This might have been due to the involvement of molecular breeding technology. Advances in molecular marker development have meant that QTLs associated with yield-related traits can be traced and used more efficiently. It is worth noting that the excellent *Hap-CC-CC-TT* haplotype selection effect is still relatively low and that *qSl-2B* still has great application potential in future breeding programs.

In conclusion, we remapped *qSl-2B*, a major QTL for SL and its genetic effects on yield-related traits were characterized using two different mapping populations. The breeding selection effect analysis indicated that *qSl-2B* had great application potential in future breeding programs. An InDel molecular marker in the target region was also developed and provided new molecular marker resources for wheat molecular breeding programs that aimed to genetically improve yield potential.

### Electronic supplementary material

Below is the link to the electronic supplementary material.

Supplementary Material 1

## Data Availability

All data supporting the results of this study are available within the paper and its supplementary data published online.
